# Terminology Matters: Advancing Science to Define an Optimal Pulse Intake

**DOI:** 10.3390/nu14030655

**Published:** 2022-02-03

**Authors:** Diane C. Mitchell, Alison Webster, Becky Garrison

**Affiliations:** 1 Diet Assessment Center, Department of Nutritional Sciences, The Pennsylvania State University, University Park, State College, PA 16802, USA; 2 Food Directions LLC, 4806 Westward View Road, Shady Side, MD 20764, USA; awebster1124@gmail.com; 3 USA Dry Pea & Lentil Council, American Pulse Association, Moscow, ID 83843, USA; bgarrison@usapulses.org

**Keywords:** pulse, beans, peas, chickpeas, lentils, legumes

## Abstract

Confusion around the terms “legumes” and “pulses” has been a long-standing problem among consumers, health professionals, and researchers in the United States. The Food and Agricultural Organization defines pulses as legumes that are harvested solely as dry grain and include beans, peas, chickpeas, and lentils. For the first time ever, the 2020–2025 Dietary Guidelines for Americans recognized and used the terminology “pulses.” Correct terminology usage is important to build a solid research foundation that is specific to pulses, primarily because of their unique nutritional attributes that impact health differently than other legumes. Future widespread conformity and standardized use of a definition and categorization system around pulses versus legumes in research would allow for an improved interpretation of science and a better understanding of current research gaps. Clarity around these gaps could enhance and improve dietary recommendations, including the ability to refine our current understanding of the optimal daily or weekly intake of pulses at which health benefits are maximized.

## 1. Introduction

The improper usage of and confusion around the terms “legumes” and “pulses” has been a pervasive problem among consumers, health professionals, and researchers in the United States [1,2,3]. Even the primary source of federal dietary advice, the Dietary Guidelines for Americans (DGA), used the term “legumes” for decades where the term “pulses” would have been more accurate. However, for the first time, the 2020–2025 Dietary Guidelines for Americans [4] used the terminology “pulses,” which they defined as the dried edible seeds of legumes including beans, peas, and lentils. The name of the vegetable subgroup previously called the “legumes (beans and peas)” subgroup was also changed to the “beans, peas, and lentils” subgroup in order to better reflect the foods included in the subgroup. Prior iterations of the DGA commonly referred to this food group as “legumes (beans and peas)” with no other clarification or references to the term “pulses” [5]. In a recent article by Didinger and Thompson [3], the ambiguity over terminology and the importance of understanding that pulses are a distinct subclass of legumes both nutritionally and functionally is described. There is little doubt that global harmonization of the terminology would allow for clearer dietary messages that may have significant public health impact, but the terminology is also important to build a solid research foundation that is specific to pulses, primarily because of their unique nutritional attributes that impact health differently than other legumes. The main objective of this communication is to demonstrate how widespread conformity and standardized use of the definition and language around pulses in research would have a broader bearing on the interpretation of the science and lead to a better understanding of the significant research gaps. Clarity around these gaps could enhance and improve dietary recommendations, including the ability to refine our current understanding of the optimal daily or weekly intake of pulses at which health benefits are maximized.

## 2. Pulses as a Distinct Class of Legumes

Although they are frequently used interchangeably, the terms “legume” and “pulse” have distinct meanings. The term “legume” broadly includes any edible parts of plants in the Fabaceae and Leguminosae family, including the leaves, stems, pods, and seeds of the plant. When used generally, this term encompasses all types of legumes including soybeans, peanuts, fresh green beans and peas, and pulses. Legumes are often split into two subcategories: oilseed legumes (peanuts and soybeans) and non-oil seed legumes. Non-oil seed legumes include pulses and freshly harvested garden vegetable varieties of legumes such as green peas, snap peas, and green beans, which are mostly consumed whole including both the pod and seeds. “Pulse” is the term used specifically for describing non-oilseed dry, nutritionally dense, edible seeds of legumes and includes dry beans, dry peas, chickpeas, and lentils. Unlike other legumes, pulses remain on plants to dry before harvesting [6]. The Food and Agriculture Organization recognizes 11 types of pulses: dry beans, dry broad beans, dry peas, chickpeas, cow peas, pigeon peas, lentils, Bambara beans, vetches, lupins, and pulses nes (‘not elsewhere specified’ or minor pulses that do not fall into one of the other categories). Commonly consumed pulses in the U.S. [7] include dry beans such as pinto, black, and kidney beans and dry peas (e.g., yellow or green peas), chickpeas, and lentils, but there are hundreds of varieties of pulses (e.g., mung beans, lupin, cowpeas, and fava beans) produced and consumed globally [6].

When conducting and reporting research regarding the health or nutritional benefits of legumes, it is important to specify the type of legume used in the study because each type has different nutritional properties. For example, oilseed legumes (peanuts and soybeans) are higher in fat and lower in dietary fiber than other types of legumes such as green beans, green peas, or pulses. Due to their dried state, pulses have a unique nutritional profile. Pulses contain about 7 to 10 g of protein per serving (~100 g or ½ cup cooked) depending on the pulse type [8,9]. They also comprise 50–65% carbohydrate including resistant starch, soluble, and insoluble fiber and have a low glycemic index. Pulses contain a vast array of phytochemicals and other bioactive components [10]. They are highly nutrient dense and, as such, are considered a significant dietary source of many nutrients such as complex carbohydrate, protein, fiber, folate, iron, magnesium, and potassium and are a good source of many other nutrients such as choline, zinc, selenium, phosphorus, and thiamin [8,9]. Significant improvements in nutrient intakes at levels around 100 g or ½ cup cooked per day of pulses have been observed in several population-based studies [7,11,12,13,14].

Despite the significant nutritional differences between pulses and other legumes, “legumes” is the terminology more commonly used in the research literature to describe pulses, even if other legumes such as soy and peanuts are excluded from consideration. There is substantial ambiguity in legume and/or pulse research since the definitions and types of legumes vary widely among studies and the term “pulses” has not been commonly used to describe this type of legume despite their clear distinction from the broader legume class.

## 3. Strengthening Pulse Research by Using Less Ambiguous Terminology

### 3.1. Prospective Cohort Studies and Randomized Controlled Trials

There is considerable evidence from observational or prospective cohort studies and randomized controlled trials (RCTs) showing the consumption of pulses is associated with positive health outcomes, such as reduced risk factors for cardiovascular disease, hypertension, diabetes, overweight or obesity, and colorectal and prostate cancers [8,15,16,17,18,19,20,21]. Several systematic reviews and meta-analyses (SRMA) have been conducted that summarize empirical evidence from many prospective cohort studies and RCTs including the dose or amount of legumes or pulses that elicit a particular response or health outcome [15,16,17,18,19,20,21]. Much of this research, however, could be strengthened by using less ambiguous terminology or by conducting research that focuses solely on pulses rather than the broader class of legumes.

In 2017, a review of SRMAs by Viguiliouk et al. [20], for example, included both prospective cohort studies and RCTs that examined the effect of dietary pulse or legume intakes on cardiometabolic outcomes. At that time of this review, there were no SRMA-conducted targeting pulses specifically; thus, data analysis included SRMAs of studies that included all legumes (pulses, soybean, soy products, peanuts, fresh peas, and fresh beans). In these prospective cohort studies, for SRMAs that included all legumes and not only pulses, a reduced risk of coronary heart disease was observed at legume intakes of four >100 g servings of pulses per week. For other outcomes such as cardiovascular, diabetes, and stroke risk, the associations remain uncertain and require further study. For RCTs where the focus was specifically targeting pulses (only dry beans, peas, lentils, and chickpeas), a decrease in cardiometabolic risk facts (e.g., HbA1c and LDL-cholesterol and body weight) at pulse intakes of 120–132 g/day (0.5–0.75 cups/day, the equivalent of about one serving/day) was observed. In one of the SRMAs reviewed, a reduction in blood pressure was achieved with a relatively high average dose of ~162 g/day (0.8 cups per day) of pulses, an amount well above current recommendations [19].

In a more recent umbrella review of SRMAs using prospective cohort studies, the associations between legumes or pulses and cardio metabolic disease outcomes were investigated. Study results showed that pulses with or without other legumes were associated with a decreased incidence of cardiovascular disease, coronary heart disease, hypertension, and obesity when comparing the highest intake of pulses with the lowest intake [21]. Although the studies specified that the exposure was “legumes,” the legumes were not differentiated by type or included types other than pulses such as soy beans, soy products, peanuts, fresh green peas, or fresh beans. However, the authors explain that the indirectness of exposure was not considered in the analysis since >50% of the studies included were from countries (mostly from Europe and North America) where pulses are consumed in much larger quantities than soy or soy products. The assumption is that the associations observed were likely due mostly to pulse intake; however, more directed research on pulses without other legumes would remove the uncertainty in estimated exposure. Moreover, associations were assessed by comparing lowest to highest quantiles of intake; thus, optimal doses of pulses were difficult to ascertain since there was a wide range of intakes when comparing the lowest to highest levels of intake.

Both epidemiological and clinical trial evidence point towards positive health benefits of consuming pulses; however, epidemiological evidence could be more directed specifically at pulses; many of the studies include other legumes or use the terminology “legumes” when it would really be more correct and specific to use the terminology “pulses.” Differing dietary exposures (e.g., pulses with and without other legumes, various pulse types, and pulse flours) make it difficult to compare and combine studies. Prospective cohort studies rely mostly on food frequency questionnaires; therefore, dietary exposures are only semi-quantitative and, thus, only an estimate of dietary (pulse) exposure is possible, making it difficult to ascertain an optimal dose for a specified effect [15,16,20,21]. In recent years, there has been significant advancement in the availability of innovative pulse products, such as pulse pastas, pulse veggie patties, and pulse flours. As these innovative products continue to emerge and consumers begin to more frequently include them in the diet, there is a need for consideration as to how these will be captured in food frequency questionnaires and other dietary survey methods in the future. Furthermore, vegetables including pulses are consumed in food mixtures as often as they are eaten separately [4,7]. Quantifying pulses using standard recipes for food mixtures may contribute to over or underestimating pulse consumption as they are largely dependent on the accuracy of the food and nutrient composition databases and self-reported estimates of intake. Consumers often are unaware of specific ingredients in the foods they consume including pulse type or amount. Advancing the science in this area requires better dietary data collection methods and improvements in databases to correctly classify, quantify, and more accurately capture pulse or legume type, all of which are impacted by how researchers and consumers define pulses or legumes.

### 3.2. Population-Based Dietary Surveys

Population-based data from dietary surveys constitute another way legumes and pulses have been studied [7,11,12,13,14,22]. Dietary surveys are used to inform dietary recommendations including DGA by providing data on trends in intake, dietary patterns, and nutrient intakes. They are also used for monitoring dietary intakes in the population and to guide research; however, most studies have focused on the broader category of legumes with little to no data on pulses specifically.

In 2009, the National Health and Nutrition Examination Survey (NHANES) was one of the first to assess intakes of pulses in the U.S. [7], with several others that followed including studies conducted on the Canadian Community Health Survey to describe pulse intakes in the Canadian population [11,12,13,14,15]. The most important and consistent finding that emerged from these few population-based studies, however, is that that diet quality is improved on days when pulses are consumed [7,11,12,13,14]. This improvement in diet quality is notable when ½ cup (~100 g) or more of pulses are consumed, and significantly higher intakes of many nutrients, especially fiber, are observed at all levels of consumption when comparing pulse consumers with non-consumers [7,11,13]. The Canadian study takes this one step further and evaluated the diets in comparison to dietary reference intakes for many of the nutrients; the results showed that a greater proportion of the population met the reference intake on days when pulses were consumed compared to days when they were not [13]. These data suggest that not only is dietary quality better on days when pulses are consumed but pulse consumers were more likely to meet dietary intake recommendations for several key nutrients.

To our knowledge, there is little or no pulse consumption data similar to the population based dietary surveys in the U.S. or Canada. This is a significant research gap, especially when considering the nutritional implications in countries where nutrient intakes are well below recommended levels. What little is known about global pulse intakes, particularly in low-income or middle-income countries, comes from per capita supply or the availability of pulses for use as food as an estimate of the average consumption but does not provide any useful data on the impact of pulse consumption on nutrient intakes or diet quality [9].

Even though dietary surveys can provide critical data for monitoring nutrient intakes, estimating intakes of specific foods, and identifying dietary patterns, they have similar limitations to other types of epidemiological pulse or legume research (e.g., prospective cohort studies) including reliance on self-reported dietary intakes, as discussed above. As with other types of pulse research, clearer definitions and classification of pulses and legumes could also improve the estimation of pulse intake from these surveys.

## 4. Categorization of Pulses and Optimal Intake

Without a doubt, the inclusion of the terminology “pulses” defined as dry beans, peas, and lentils in the 2020–2025 DGA is a step towards recognizing the unique characteristics of pulses separately from other legumes; however, there is still room for more clarity in the DGA [4]. For example, green soybeans (edamame) are included in the vegetable subgroup “beans, peas, lentils” despite being the only non-pulse included in the subgroup. Green (snap) peas and green beans (string beans/snap beans) are both legumes, but green peas are placed in the “starchy vegetable” subgroup and green beans are placed in the “other vegetable” subgroup even though they have similar compositions. DGA also do not include chickpeas often referred to as garbanzo beans as a separate pulse type in their definition of a pulse, which could also contribute to confusion as it does not recognize chickpeas as the separate botanical group it is and does not fully align with the FAO’s definition of a pulse [6].

When considering individual dietary patterns in the most recent DGAs, foods in the “beans, peas, lentils” subgroup can be considered either a vegetable or a protein to meet recommended intakes [4]. Despite some confusion in the U.S. guidelines, this is even more persistent in dietary guidance across the globe even though the terminology “pulse” is more commonly used and understood [23]. The inconsistency is really in the placement of pulses in the diet; for example, some countries place pulses in a “fruit and vegetable” category while others place pulses in a “meat and meat alternate” category. Much of these inconsistencies might be a reflection in differences in dietary patterns or in the manner pulses are usually consumed [23]. For example, in some African and Asian countries, pulses are a staple food consumed almost every day, whereas in the U.S., Canada, and Europe, pulses are more typically consumed as an occasional side dish or soup and often in place of other vegetables [7,24].

There is also little consensus globally about what constitutes a pulse serving size or the recommended frequency of pulse consumption [23]. In the U.S., there has been some rather significant changes in the recommendations over time [4,24,25]. The most recent 2020–2025 DGAs recommend 1.5 cup equivalents of beans, peas, and lentils per week for individuals following a 2000-calorie healthy U.S.-style dietary pattern or a 2000-calorie healthy Mediterranean-style dietary pattern. For individuals following a 2000-calorie healthy vegetarian dietary pattern, the DGAs recommend 3 cup equivalents of beans, peas, and lentils per week [4]. The 2005 DGA [25] specified 3 cups of legumes (beans and peas) per week as the recommended level of intake for all individuals (i.e., not only one subset of individuals such as vegetarians) based on their contributions of nutrients that were limiting in the diets of Americans (e.g., potassium, folate, and fiber); however, in 2010, this recommendation decreased to 1.5 cups of legumes per week with no apparent justification [24]. There is a rather complex history of how DGAs have evolved over time with a shift towards more science-based recommendations and, perhaps, this explains to some extent why recommendations for pulses (or legumes) have changed [26]. With that in mind, there is little doubt that scientific evidence will be the basis for any change in current recommendations. While limited in scope, the more recent literature that is available has suggested that positive health outcomes are observed at pulse intakes of around ½ cup or 100 g per day, which is higher than the amount currently recommended by the DGA for individuals following a healthy U.S.-style and healthy Mediterranean-style dietary pattern. More directed research on pulses specifically using consistent pulse terminology and better agreement about where pulses fit into the context of a healthy dietary pattern would provide a stronger basis for defining optimal intakes for health and ensure dietary guidance is recommending that American’s consume the most optimal amounts.

## 5. Future Directions

Despite the research gaps and limitations of the existing literature, population-based consumption data show improvements in diet quality on days when pulses are consumed [7,11,12,13,14]. Other epidemiological evidence demonstrates associations between pulse intake and health outcomes and clinical evidence shows positive metabolic response to diets containing pulses [15,16,17,18,19,20,21]. Furthermore, the studies support health benefits from consuming at least ½ cup cooked (~100 g) of pulses per day, which suggests that intakes above 1.5 cups per week may be more optimal [7,11,13,20]. Less than 10% of the U.S. population meets recommendations for fiber, and many of the key nutrients in pulses such as potassium, magnesium, and choline were noted by the DGA 2015 committee as nutrients of concern [5]. This alone supports increasing pulse consumption as one potential strategy towards achieving healthier dietary patterns. Another potential strategy is shifting towards more plant-based diets by substituting some or most of the animal protein with plant sources such as pulses. This is often referred to as a flexitarian approach and is increasingly popular for its perceived health benefits, but more research on flexitarian diets that incorporate pulses is needed. Our market place continues to expand with a plethora of innovative ways pulses are incorporated into a variety of foods. The unique composition of pulses makes them well suited for incorporation into pastas, cereals, snack foods, soups, and beverages. These innovative food technologies could play a role in overcoming hurdles to increase pulse consumption making pulse-based food more palatable and nutritious.

Additional research specifically directed at pulses apart from the broader legume category is warranted. Long-term clinical trials assessing pulse exposure within well-controlled diets will add to the body of evidence around the optimal intake of pulses at which health benefits are maximized. Future research from national food intake surveys including modeling studies that quantify the impact of pulse ingredients as additions and/or replacements in multi-component foods will be important to further capture the impact of pulses on nutrient intakes from these foods. Modeling studies rely on the placement in the context of a healthy diet (e.g., pulses can be counted as both a vegetable or a protein) and how pulses are defined. Advancing the science in this area requires better data collection methods and improvements in databases in order to more accurately capture and quantify the intake of specific pulses. Identifying and correctly classifying legumes and pulses will advance all types of research in this area, refine dietary recommendations, and improve public health.

## Data Availability

Not applicable.
